# T lymphocytes and natural killer cells in myelodysplastic syndromes: function, dysfunction, and therapeutic potential

**DOI:** 10.3389/fimmu.2026.1907953

**Published:** 2026-09-04

**Authors:** Justin Cheng, Eric Leon Tam, Casey O’Connell

**Affiliations:** 1 Keck School of Medicine, University of Southern California, Los Angeles, CA, United States; 2 Jane Anne Nohl Division of Hematology and Center for the Study of Blood Diseases, University of Southern California, Los Angeles, CA, United States

**Keywords:** hypomethylating agents (HMA), immune checkpoint inhibition, immunosuppressive therapy, MDS, T lymphocytes

## Abstract

Myelodysplastic syndrome (MDS) are clonal myeloid neoplasms that cause cytopenias and can progress to acute myeloid leukemia (AML). Hypomethylating agents (HMA) are the mainstay of treatment for higher risk disease, but they achieve responses in only half of treated patients and complete remission rates are low. Several scientifically based combinatorial regimens have been tested in clinical trials but none has demonstrated a survival benefit over HMA monotherapy. Allogeneic stem cell transplant remains the only curative therapy and is dependent on effective donor lymphocytes for its efficacy. However, access is limited by its toxicity, so alternative approaches are sorely needed. Recent clinical and translational studies have shown that MDS is not only a clonal myeloid disorder, but also associated with immune dysregulation, inflammatory signaling, T-cell repertoire restriction, immune exhaustion, and immune mediated suppression of hematopoiesis. These findings suggest that there is potential for unlocking a novel approach to the treatment of MDS by restoring and/or enhancing the lymphoid immune response. In this review, we discuss the current understanding of the role of normal T lymphocytes in MDS, the causes and manifestations of dysfunctional T lymphocytes as well as the role and dysfunction of natural killer (NK) cells. The role of therapeutic immunosuppression in lower risk MDS is reviewed, as is the impact of HMAs on dysregulated T lymphocytes and NK cells in higher risk disease. We propose that there is tremendous potential for more targeted approaches to engage the lymphoid compartment in addressing the unmet therapeutic need in MDS.

## 1 Background

Myelodysplastic syndromes (MDS), now known as myelodysplastic neoplasms in the most recent classification systems 5^th^ Edition WHO (1) and ICC (2), are a heterogeneous group of myeloid malignancies characterized by ineffective hematopoiesis with recurrent genetic abnormalities and morphologic dysplasia, resulting in cytopenias and variable increase in risk of progression to acute myeloid leukemia. The clinical presentation of MDS also varies significantly, ranging from indolent cytopenias to highly proliferative and genomically complex disease that portends a high risk for leukemic transformation (3). Because of the heterogeneity in MDS, accurate risk stratification is imperative to provide management and therapy at the appropriate level of intensity. IPSS-R is still the standard on the National Comprehensive Cancer Network (NCCN) guidelines, which incorporates clinical, cytogenetic, and hematological data. Most recently, the IPSS-M module incorporates molecular and NGS data with clinically and cytogenetic variables, and has been well validated (4, 5).

Lower risk MDS treatment focuses on symptomatic control, cytopenia management, and transfusion independence. Current treatments include supportive care therapies such as erythropoiesis stimulating agents (ESA), the telomerase inhibitor imetelstat (6), and luspatercept (7), an erythroid maturation agent targeting transforming growth factor-β (TGF-β) signaling. For higher risk MDS, management is focused on disease modification, delaying AML progression, and for suitable candidates, allogeneic stem cell transplantation for definitive cure. Monotherapy with hypomethylating agents (HMA) remains the therapeutic backbone for high risk MDS, with azacitidine showing improved overall survival compared to conventional care in higher risk MDS (8). Oral decitabine-cedazuridine also demonstrated clinical activity in MDS from the ASCERTAIN study (9), but so far, no hypomethylating agent combinations have produced consistent survival benefits. Recently, precision therapy with targeted agents such as ivosidenib (10) has shown activity in refractory IDH1 mutated MDS, but many patients with MDS lack directly actional genomic targets.

Ultimately, allogeneic stem cell transplantation remains the only established curative therapy (11). However, access is limited due to patient factors such as performance status and comorbidities. Notably, transplant is an immunologic intervention whose efficacy depends on the biology of donor-derived T lymphocytes. Therefore, T lymphocytes play a critical, though incompletely understood, role in the biology of MDS, and when optimally functioning, T lymphocytes are fundamental to the only curative modality currently available.

Recent clinical and translational studies have shown that MDS is not only a clonal myeloid disorder, but also associated with immune dysregulation, inflammatory signaling, T-cell repertoire restriction, immune exhaustion, and immune mediated suppression of hematopoiesis (12–15). These findings have led to contrary approaches in targeting T lymphocytes. A subset of hypoplastic MDS with lower-risk patients display clinical and biologic features of immune-mediated marrow failure, including hypocellularity, profound cytopenias, restricted T-cell receptor repertoires, HLA associations (16), and responsiveness to immunosuppressive therapy (17). Similarly, autoimmune cytopenias that emerge in the presence of MDS have been successfully treated with immunosuppression (18).

Higher-risk disease, on the other hand, has been associated with immune exhaustion (12), regulatory T-cell expansion (19), myeloid-derived suppressor cell (MDSC) activity (20), checkpoint ligand expression, and impaired antitumor cytotoxicity. Several trials, with variable success rates, have employed immune checkpoint inhibition to stimulate T cell function in higher risk disease. Moreover, mutated immune cells may create a chronic inflammatory state that contributes to the competitive advantage and clonal dominance of MDS-derived hematopoietic cells.

In addition to T lymphocytes, dysregulated natural killer (NK) cell cytolytic activity has been observed in MDS. NK cells are mediators of innate immune surveillance against myeloid neoplasms and their cytolytic function has been shown to be impaired in MDS (21). Particularly, downregulation of the NK cell activating receptors NKp30 and NKG2D along with reduced NK cell function was observed in higher risk disease. NK cells have been shown to express PD-1 and become functionally exhausted under chronic antigenic pressure with upregulation of NK cell activity in PD-1 blockade (22).

As the cellular crosstalk between the lymphoid system and the malignant myeloid clone is systematically deciphered, the MDS treatment paradigm should dramatically expand to enable more potent and targeted engagement of the immune system. In this review, we outline the salient and less overt features of immune dysregulation involving T lymphocytes and NK cells and highlight the potential for immune-targeted therapies to help fill the unmet therapeutic need in MDS.

## 2 T lymphocytes in the bone marrow microenvironment in MDS

### 2.1 Normal bone marrow immune architecture

Characterization of the normal bone marrow (BM) microenvironment has historically been constrained by technical limitations, particularly the difficulty of isolating non-hematopoietic stromal populations (<0.5% of total cellularity) and sampling bias skewed towards adipocyte-rich populations (23). The increased use of single-cell transcriptomics and spatial mapping approaches have substantially revised this view, enabling the detailed annotation of distinct cellular BM neighborhoods that coordinate hematopoiesis (23, 24).

Hematopoiesis is spatially compartmentalized into functional niches rather than occurring within a uniform marrow milieu (25). Spatial mapping studies have defined an atlas of bone marrow neighborhoods, each with distinct cellular and functional components (23): an arterio-endosteal niche for early myeloid and granulocyte-monocyte progenitors, an adipocyte niche where HSPCs lie close to adipocytes and adipogenic mesenchymal stem cells (MSC), a sinusoidal niche associated with mature myeloid differentiation, and a peri-arteriolar lymphoid niche enriched for T and B cells (26, 27). With age, this organization is progressively remodeled, with expansion of adipocytes, reduced osteolineage support, and increased inflammatory tone. Inflammatory and infectious states can further alter niche organization, promoting myeloid skewing (28), reducing lymphopoiesis, and creating a permissive baseline for clonal selection (29).

Among these regions, the peri-arteriolar lymphoid niche is spatially and functionally distinct from the HSC-supporting regions. MSCs within this niche are a major source of CXCL12 and IL-7 that serve to support naïve T cells (30). Arteriolar endothelial cells in this niche provide Notch ligands that contribute to lymphoid commitment and maintenance (31). As a result, disruption of the niche and these spatial signals is expected to also alter T cell composition and function even before direct exposure to the myeloid clone.

### 2.2 Niche disruption and spatial remodeling in CHIP and MDS

Clonal hematopoiesis of indeterminate potential (CHIP) is increasingly recognized as a principal driver of the early remodeling of the niche architecture (29). Many of these CHIP-associated driver mutations have independently been associated to promote a pro-inflammatory BM environment through distinct mechanisms. For example, TET2 loss of function in myeloid cells leads to sustained IL-6 overproduction through the loss of HDAC2-mediated transcriptional repression (32). CHIP-mutated myeloid cells have demonstrated amplified NF-kB activation and exaggerated inflammatory response to pattern recognition receptor stimulation (33). This inflammatory conditioning may provide a selective pressure for further clonal expansion and establish a low-grade inflammatory marrow environment.

This early remodeling is qualitatively distinct from the niche disruption in MDS. Single-cell and spatial profiling of BMs across the continuum has demonstrated that iMSCs first emerge during CHIP and become progressively more prevalent in MDS. However, the functional consequences of this remodeling still differ, with co-culture CHIP-derived HSPCs maintaining robust stromal support programs through CXCL12, CSF1, and GM-CSF at similar levels to healthy controls, whereas MDS-derived HSPCs fail to sustain these programs (34). As a result of the stromal loss of homeostatic CXCL12+ MSCs, the aforementioned peri-arteriolar lymphoid niche that sustains naive T cell pools is disrupted (35), establishing the conditions for the T-cell dysfunction and spatial remodeling elaborated below.

A defining early event in MDS BM remodeling is the progressive loss of homeostatic CXCL12+ MSCs and their replacement by an inflammatory MSC compartment (36, 37). These inflammatory MSCs frequently exhibit activation of canonical inflammatory pathways in NF-kB and IL6 signaling and show reduced ability to support healthy hematopoiesis. MDS MSCs also demonstrate pathological overexpression of CXCL12 in a spatially disorganized pattern. In the healthy marrow, CXCL12 is largely restricted to perivascular regions. However, MSCs in MDS and AML lose their specific homeostatic CXCL12+ niches that support normal HSPCs and instead secrete CXCL12 broadly, which may tether malignant cells and promote their retention in abnormal niches (38). MDS patients responding to HMAs were seen to have lower CXCL12 expression at remission compared to pre-treatment, suggesting that stromal dysfunction is correlated to disease activity (39).

A key driver of this remodeling is the signaling between dysplastic HSCs and T cells (35). Activated T cells responding to dysplastic HSCs secrete proinflammatory cytokines that reinforce the inflammatory rewiring of MSCs. Several explanations have been proposed for blast persistence in this niche, including dysregulation of inflammatory signaling pathways in IFN-γ and TNFα (40) and upregulation of anti-apoptotic BCL-2 family members in MDS clones (41). In either case, the result is a niche that selectively suppresses normal HSPC repopulation, while mutant HSPCs demonstrate relative resistance to this stress (35).

Lessons from AML and MDS cellular and immunotherapy have suggested that the BM spatial organization of immune cells may also influence response to therapy. In relapsed AML treated with donor lymphocyte infusions, responders exhibited more diverse baseline peri-arteriolar lymphoid niches, while non-responders showed myeloid-dominant neighborhoods with reduced cellular heterogeneity (42). Responders further shifted towards cytotoxic effector phenotypes whereas non-responders showed enrichment of an exhausted T cell phenotype (42). In MDS, abnormal CXCL12 secretion was associated with increased malignant cell retention in stromal niches while T cells remained in more distant peri-arteriolar lymphoid compartments, creating a physical separation between cytotoxic T cells and malignant cells (36). Whether stromal remodeling directly improves active CD-8 T cell access to the malignant compartment remains to be formally demonstrated but offers a spatially coherent explanation.

MDS is a disease of both the malignant clone and its immune niche that allows for clonal persistence. The spatial position of T cells relative to malignant cells, the stromal context, and the cellular diversity of their local neighborhood are all relevant to immune dysfunction and therapeutic response, and larger prospective trials that incorporate spatially resolved BM architecture following HMA and combinatorial treatment are needed.

## 3 Clinical evidence of T cell dysregulation and therapeutic implications

MDS arises in the myeloid compartment, but also elicits an adaptive immune component that co-evolves in a chronic inflammatory marrow microsystem, resulting in bidirectional influences. The malignant myeloid clone contributes to shaping T cell biology by generating antigens, altering antigen presentation, remodeling cytokine networks, inducing checkpoint pathways, and recruiting suppressive immune populations (43, 44). Simultaneously, the T-cells influence the myeloid clone by suppressing normal hematopoiesis, exerting immune pressure against dysplastic progenitors, selecting for immune evasive subclones, and becoming exhausted as disease progresses.

One of the most consistent observations that support T-cell involvement in MDS is the presence of a skewed T-cell receptor repertoire, especially in cytotoxic CD8 positive T cells. CDR3 length typing and flow cytometric analysis of T cel receptor (TCR) Vβ families labelled with specific monoclonal antibodies are the most frequently used assays for the analysis of TCR Vβ repertoire (45). Early TCR Vβ repertoire studies found differences in T-cell populations in MDS compared to healthy controls that was consistent with antigen-driven expansion rather than random immune activation (43). Additional studies demonstrated that patients with MDS had more frequent skewing of TCR Vβ profiles than age-matched controls, indicating that T-cell-mediated immune processes is an inherent feature of MDS biology (46). Cytotoxic T cells have oligoclonal or highly contracted TCR repertoires (47), supporting the concept that chronic antigen stimulation is common in MDS.

With TCR restriction, some T-cell expansions in MDS may recognize antigens related to dysplastic hematopoiesis. Potential antigenic drivers include neoantigens generated by somatic mutations, aberrantly expressed self-antigens, spliceosome-altered peptides, leukemia-associated antigens, stress-induced antigens, or antigens associated with abnormal karyotypes. The strongest clinical examples come from studies of trisomy 8 MDS and WT1-directed immune responses. Sloand et al. demonstrated that autologous lymphocytes could preferentially suppress trisomy 8 hematopoietic progenitors compared with cytogenetically normal progenitors, supporting a model in which cytotoxic T cells can directly recognize and suppress abnormal hematopoietic clones (48). However, this T-cell response may also contribute to cytopenias by suppressing normal hematopoiesis. This duality is one of the central paradoxes of T-cell biology in MDS. The cytotoxic immune response may restrain the clone while simultaneously damaging hematopoietic function.

Inflammatory T-cell subsets also appear to differ by disease stage. Lower-risk MDS has been associated with a pro-inflammatory immune phenotype, with increased Th17 cells, inflammatory cytokines, and apoptotic signaling within the marrow compartment, suggesting that inflammatory T-cell polarization may contribute to ineffective hematopoiesis and marrow injury (49). This inflammatory state may preferentially damage residual normal hematopoietic stem and progenitor cells while allowing mutant clones with inflammatory resistance or survival advantages to persist, serving as a selective pressure that promotes clonal dominance. As MDS progresses, the T-cell landscape shifts from inflammatory surveillance toward immune dysfunction and immune escape. Regulatory T cells are increased in higher-risk MDS and have been associated with progression to more aggressive disease. The expansion of CD4-positive CD25-high FoxP3-positive regulatory T cells has been shown to be a feature of high-risk MDS and associated with progression (19). This suggests that the immune system in MDS transitions from a relatively inflammatory, cytotoxic, marrow-suppressive state in lower-risk patients to a more immunosuppressive and tumor progressive state in advanced disease.

MDSCs cells add to this immunologic transition. These suppressor populations inhibit T-effector cell proliferation and cooperate with regulatory T cells to impair antitumor immunity. MDSCs from patients with MDS have been shown to suppress T-effector proliferation and MDSC levels correlate with regulatory T-cell numbers in higher-risk disease (20). MDSCs have been seen to be markedly expanded in the BM of MDS patients and correlated with disease risk and progression (50). Co-culture of MDSCs with CD8+ T cells resulted in the induction of T cell apoptosis and decreased perforin and granzyme production (51). In a recent trial of decitabine combined with ATRA vs decitabine alone in 277 MDS-EB patients, treatment with the combination demonstrated a significantly higher ORR (78% vs 51%), reflecting that ATRA-mediated myeloid differentiation may complement HMAs (52). Though this trial did not specifically measure MDSC depletion, ATRA has been previously demonstrated to force MDSC maturation with promising results in solid tumors (53), and whether this contributes to HMA sensitization in MDS remains unclear. The negative phase III SELECT-MDS-1 trial of tamibarotene, a selective RARα agonist, may then be partially explained by tamibarotene’s RARα selectivity (54). Early phase studies specifically targeting MDSCs with AMV564, a CD33/CD3 bispecific T cell engager, demonstrated improvement in hematopoiesis following MDSC depletion and subsequent activation of cytotoxic T cell function (55). However, further development has been discontinued following the company’s closure, though the early signal is suggestive that MDSC targeting may be feasible in MDS.

Recently, immune checkpoint pathways have suggested an additional mechanism through which the MDS clone may modulate T-cell function. PD-1/PD-L1 signaling, CTLA-4, TIM-3, TIGIT, and other inhibitory pathways have been studied in MDS and related myeloid neoplasms. Preclinical and translational studies have shown that MDS cells can upregulate PD-L1 in response to tumor-specific T-cell activity, demonstrating that an initially active immune response may induce adaptive immune resistance (56). Furthermore, high-risk MDS cytotoxic T cells have been reported to have reduced cytotoxic capacity (57), demonstrating impaired T-cell effector function contributes to ineffective immune surveillance in advanced disease.

These findings links the malignant or premalignant myeloid compartment to active suppression of adaptive immunity; the myeloid clone does not evade T cells passively but actively builds an immune microenvironment that disables effective T-cell responses.

**Figure 1:**
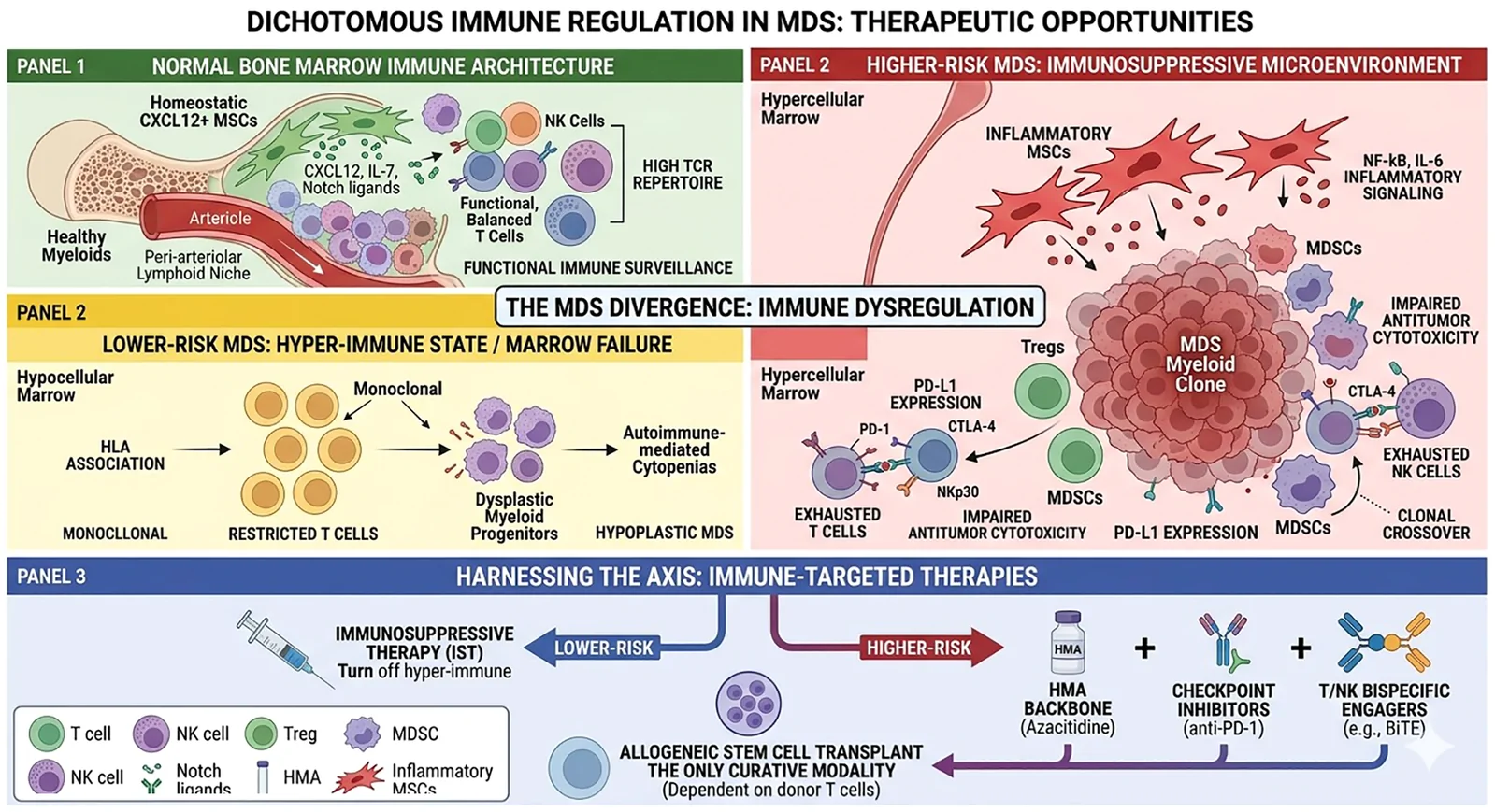
Dichotomous immune regulation in myelodysplastic syndromes and associated therapeutic opportunities. Panel 1 depicts normal bone marrow architecture, including homeostatic mesenchymal stromal cell signaling, balanced lymphocyte populations, and intact immune surveillance. Panel 2 depicts lower-risk MDS, characterized by restricted T-cell repertoires and immune-mediated marrow failure with higher-risk MDS characterized by inflammatory signaling, regulatory T-cell and myeloid-derived suppressor cell expansion, checkpoint activation and T- and NK-cell exhaustion. Panel 3 summarizes stage-adapted immune-directed strategies, including immunosuppressive therapies in selected lower-risk disease and hypomethylating agents, immune checkpoint inhibitors, bispecific engagers, and allogeneic stem cell transplantation in higher-risk disease. BiTE, bispecific T-cell engager; CTLA-4, cytotoxic T-lymphocyte-associated protein 4; HLA, human leukocyte antigen; HMA, hypomethylating agent; IFN, interferon; IL, interleukin; MDS, myelodysplastic syndrome; MDSC, myeloid-derived suppressor cell; MSC, mesenchymal stromal cell; NF-κB, nuclear factor kappa B; NK, natural killer; PD-1, programmed cell death protein 1; PD-L1, programmed death ligand 1; TCR, T-cell receptor; TNF, tumor necrosis factor.

Given these observations, treatment of MDS requires an understanding of the underlying T- cell state as the same compartment plays opposing roles depending on disease stage (**Figure 1**, **Table 1**). As earlier discussed and in prior reviews, T-cell activity in lower-risk MDS has been linked to suppression of normal hematopoietic progenitors for which immunosuppression may rescue normal hematopoiesis (35, 58). As disease progresses to higher-risk categories, Treg expansion and exhaustion markers accumulate, and activation of T-cell function through epigenetic reprogramming or checkpoint activation may allow for greater clinical benefit. Scoring systems such as the IPSS-R/IPSS-M remain the main risk-stratification tool for MDS, though are limited in their ability to capture T cell and immune dysfunction. The MDS-Immune-Risk (MIR) score was among the first to incorporate specific immune-risk factors beyond IPSS-R, with high MIR scores predicting inferior OS independent of IPSS-R category and somatic mutations (59).

**Table 1:** Stage-dependent T-cell and NK-cell immune abnormalities in MDS.

Immune abnormality	Predominant disease stage	Mechanism/functional consequence	Potential therapeutic implication
T cell abnormality			
Inflammatory T-cell activation (Th17 expansion, ↑IFN-γ, ↑TNF-α)	CHIP, CCUS, lower-risk MDS	Inflammatory cytokines suppress normal hematopoiesis while creating selective pressure for resistant clones	Immunosuppressive therapy (selected patients); anti-inflammatory approaches under investigation
Oligoclonal CD8+ T-cell expansion / restricted TCR repertoire	Lower-risk MDS and hypoplastic MDS	Antigen-driven cytotoxic T cells suppress normal HSPCs more than the malignant clone, creating a selective pressure and producing cytopenias	IST (ATG/cyclosporine) in selected patients; TCR repertoire/HLA-DR15 may serve as biomarkers for response
LGL-like cytotoxic T-cell expansion (STAT3/STAT5B-mutated clones)	Lower to intermediate-risk MDS with LGL overlap	Pathogenic cytotoxic T-cell clone drives lineage-specific cytopenias independent of the myeloid clone	LGL-directed therapy (methotrexate, cyclophosphamide); sorted STAT3/STAT5B sequencing
Regulatory immune suppression (Treg expansion, MDSC accumulation)	Intermediate to higher-risk MDS	Suppression of antitumor immunity through Treg inhibition and MDSC-driven T cell apoptosis; myeloid clone actively creates immunosuppressive microenvironment	HMAs (partial Treg reduction in responders); ATRA + HMA (MDSC directed differentiation/ROS-dependent cytotoxicity); MDSC targeted bispecifics (early stage)
Checkpoint activation and T-cell exhaustion (PD-1, PD-L1, TIM-3, CTLA-4)	Higher-risk MDS, post-HMA exposure	Adaptive immune resistance in response to T-cell pressures; terminal exhaustion renders T cells refractory to single agent checkpoint reinvigoration	Limited studies demonstrating benefit. HMA + ICI in treatment naïve, limited benefit in HMA-failure patients (possible benefit in biomarker stratified cohorts). TIM-3 inhibition and dual ICI intensification with limited efficacy
NK cell abnormality			
NK-cell dysfunction (↓NKG2D, ↓NKp30, ↓KIR diversity, impaired degranulation)	Progressive throughout disease continuum; most pronounced in higher-risk MDS	Reduced innate immune surveillance and impaired leukemic cell killing	HMAs partially restore NK function; emerging NK-cell–directed therapies under investigation

More recently, an immune dysfunction score (IDS) derived from single-cell transcriptomic profiling was validated in a multicenter cohort of 432 MDS patients, with IDS-high cases demonstrating depletion of cytotoxic T-cell and dendritic cell signals not captured by conventional response criteria. The authors then integrated IDS with drug response data from the BeatAMLv2 dataset, with the results suggesting that IDS-high cases were preferentially vulnerable to multikinase and NF-kB pathway inhibitors (60). Trials using NF-kB inhibitors such as bortezomib have shown modest clinical efficacy, though have focused on lower-risk MDS patients and may show greater benefit then in patients with an anti-apoptotic BM phenotype, and further prospective validation of IDS-guided therapeutic selection is still needed (61). Markers that predict when MDS patients may benefit from immune suppression versus immune activation are needed as risk category alone may be insufficient to make that determination. Standardization of these immune monitoring parameters across MDS trials, as outlined by the i4MDS consortium, will be essential to validate whether these immune scores can guide therapeutic selection (62).

## 4 T-cell involvement by “the clone”

Clonal T cell expansions are seen in approximately half of MDS patients and have been seen to be preferentially enriched in the bone marrow compared to the peripheral blood (63). These expanded T cell populations are primarily CD8+ and display a terminally differentiated CD57+ CD28- phenotype (64). While this expansion can represent anti-tumor responses, they are frequently functionally impaired and may contribute to bystander suppression of normal hematopoiesis. What is less clear is whether these clonal or oligoclonal T cell populations or NK cells have acquired the same mutations as the myeloid clone(s).

In a systematic analysis of sorted CD3+ T lymphocytes from 27 patients with MDS previously treated with HMAs, our group detected involvement by myeloid driver mutations in the lymphoid compartment (using a cutoff of >20% of cells) for several genes (65). Among epigenetic regulators, T cell mutations were detected in 5/12 with ASXL1, 4/7 with *EZH2*, 2/6 with *TET2* and 3/5 with *DNMT3a*. The variant allele frequencies of the mutations in the T cells were lower than in the myeloid compartment, as would be expected if we assume the myeloid mutation preceded T cell involvement. Of particular interest was that the only patients to achieve complete remission with the combination of an HMA with an immune checkpoint inhibitor in this study harbored co-mutations (mutations in both myeloid and lymphoid compartments) in *ASXL1*, suggesting that lineage-specific mutation patterns may provide the potential for personalized immune therapy, discussed further in Sections 7.1 and 8.

Systemic inflammatory and autoimmune disorders (SIADs) occur in 10-20% of patients with MDS (66) and in 15-28% of patients with CMML (67) and have stimulated interest in the clonality and function of T- and NK cells in these myeloid malignancies. Molecular studies indicate that mutations in *TET2*, *IDH1/2*, and *SRSF2* are more frequently associated with SIADs in patients with MDS or CMML and that *TET2* and *IDH* mutations affect T cell function in a similar manner, suppressing the CD8+ T cell memory population (68). Boy et al. demonstrated *TET2* mutations in peripheral blood natural killer (NK) cells but not T lymphocytes. In the mutated NK cells there was an inverse correlation between the variant allele frequency (VAF) of the *TET2* mutation and expression of certain killer immunoglobulin-like receptors (KIRs) leading to reduced NK cell function in the cells with a higher mutation burden. The authors further demonstrated *in vitro* as well as with post-treatment samples from patients with *TET2*-mutated MDS that hypomethylating agents led to restoration of KIR expression on NK cells (69). As TET2 is among the most frequently mutated genes in CHIP-associated HSPCs (70), NK cell immune surveillance may already be compromised at the CHIP stage well before overt MDS. As such, NK cell profiling should be considered in prospective CHIP cohort studies to define the clinical significance and timing of early immune dysfunction.

VEXAS (vacuoles, E1 enzyme, X-linked autoinflammatory, somatic) syndrome is the first monogenic disorder consistently associated with both a systemic inflammatory syndrome as well as an underlying myeloid or plasma cell neoplasm, providing potential insight into the causal relationship between SIADs and MDS. *UBA1* mutations do not appear in mature T lymphocytes (71), though they have been shown to be present in circulating NK cells in affected patients (72, 73) and to adversely affect NK cell number and function (74). Gutierrez-Rodrigues et al. observed, based on variant allele frequencies of coexisting mutations, that clonal hematopoiesis can co-occur, predate, or follow the *UBA1* mutation, making it difficult to determine the direction of causality between MDS and SIADs in the VEXAS model.

Clonal involvement by classical myeloid mutations in the lymphoid compartment is a novel concept that should be further investigated because it has the potential to expand the limited treatment options currently available to patients with MDS.

## 5 Effect of HMAs on T cell function, the TCR repertoire and NK cell function

Since their FDA approval in the early 2000s, HMAs such as azacitidine (AZA) and decitabine (DEC) have been a mainstay in treatment for patients with MDS. In both higher and lower risk MDS, HMAs have demonstrated improved rates of transfusion independence; in HR-MDS, they delay progression to AML compared to supportive or conventional care, with a mortality benefit for azacitidine in HR-MDS. These agents are incorporated into DNA or RNA and deplete DNMT1 (75), resulting in passive DNA hypomethylation and re-expression of epigenetically silenced genes. Their efficacy and relative ease of use have allowed them to serve as backbones for many combination regimens for clinical trials in MDS. However, complete responses to HMAs are limited and often non-durable, with poor outcomes following refractory disease (36).

### 5.1 HMA modulation of tumor immunogenicity

Methylation studies of MDS patients have revealed widespread epigenetic dysregulation thought to contribute to altered hematopoietic differentiation of the malignant clone (76). HMAs are able to restore immune-relevant pathways by irreversibly inhibiting DNMT, particularly DNMT1, and interfering with methylation maintenance following DNA replication. Whole-genome methylation analyses of paired MDS-control samples have demonstrated increased hypermethylation burden of CpG islands and promoter regions such as CDKN2B, ZNF300, FZD9, involved with transcriptional silencing and hematopoietic regulation (77, 78). At the same time, total genome methylation levels in MDS appear to be lower or similar to controls, with more hypomethylated than differentially methylated regions detected in genome-wide analyses (77). As such, aggregate methylation burden has not emerged as a clinically useful biomarker (79), though many specific sites have been associated with MDS pathogenesis and progression. For example, high methylation density (>50%) of BCL2L10 has been associated with reduced responses to HMAs compared to low methylation densities (80). Similar reductions in DLC-1 promoter methylation has been associated with clinical response in MDS and AML patients (81).

Another proposed antineoplastic mechanism of HMAs is the reactivation of human endogenous retroelements (ERE) and other transposable elements normally silenced by DNA methylation. EREs represent approximately 43% of the genome and consist of human endogenous retroviruses, retrotransposons, and other inactive fragments (82). HMA-mediated transcription of these elements have been associated with type I interferon responses and upregulation of IFN1 genes. This “viral mimicry” state may enhance immunogenicity and promote cytotoxic T cell recognition. In a transcriptomic analysis of HMA-treated AML/MDS cohorts, higher viral mimicry scores following HMA treatment were associated with improved responses to HMA (83). In another study of MDS patients treated with azacitidine, responders showed greater early hypomethylation of CpG sites associated with EREs compared with nonresponders. Responders later were seen to have increased expression of specific transposable element families accompanied with increase in inflammatory response pathways (84).

### 5.2 HMA effects on T cell activation and phenotype

In addition to increasing tumor antigenicity, HMAs also directly modulate T-cell function, particularly T-cell activation and exhaustion. In the CD8+ T cell compartment, low dose decitabine exposure has been associated with enhanced CD8+ T cell activation, increased cytolytic function, and greater CD8+ T cell infiltration into the tumor microenvironment (85). Interestingly, these effects occurred despite an overall decrease in CD8+ T cell expansion, and an enrichment of a specific granzyme B+/perforin+ effector subpopulation, suggesting that HMAs may specifically skew T cells more towards an activated cytotoxic phenotype. Subsequent epigenomic profiling also demonstrated upregulation of NFAT-associated cytolytic pathways involved in T-cell activation following decitabine exposure. Decitabine and azacitidine have also both been demonstrated to upregulate the expression of CD70, CD1d, and NK receptor ligands on AML blasts, enhancing NK cell-mediated cytotoxicity and complementing the CD8+ T cell response (86). In the post-transplant setting, azacitidine induced increased cytotoxic CD8+ T-cell responses against multiple tumor antigens, including MAGE-A1 and WT-1 following allogeneic SCT, and occurred concurrently with early Treg expansion (36).

In the CD4+ T cell compartment, the effects of HMAs on Tregs have been conflicting. In a study of 68 patients with intermediate- to high-risk MDS, baseline Treg frequencies were significantly elevated compared with healthy controls, consistent with an immunosuppressive marrow, and responders to azacitidine demonstrated a reduction in BM Treg frequency over time compared to nonresponders (36). Azacitidine has also reduced CD4+ T cell proliferative capacity and reduced suppressor function *in vitro*, while increasing IL-17 production. In the post-transplant setting however, HMAs were seen to simultaneously increase both CD8+ T cells and Treg numbers compared to controls, yet was also associated with CD8+ tumor directed cytotoxicity and reduced alloreactive GVHD (87). This data may be reconciled by differing levels of Treg function compared to absolute numbers. Decitabine-induced Tregs have been shown *in vitro* to exhibit incomplete suppressive function, arising primarily from conversion of CD25- T effectors rather than expansion of thymic T-regs (88). These HMA-induced Tregs may preferentially suppress broad T cell responses while leaving antigen-specific CD8+ T cell responses intact. Absolute Treg numbers in isolation are therefore unlikely to serve as a reliable biomarker of immune response following HMA treatment.

The effect of HMAs on the induction of checkpoint inhibition pathways and its effect on CD8+ T cell exhaustion is clinically well characterized. In a study of 27 MDS and AML patients, azacitidine was associated with PD-1 promoter demethylation in 44% of patients, and accompanied by increased PD-1 expression on T cells and inferior ORR (36). Increased expression of PD-L1, PD-L2, and CTLA4 mRNA levels have also been observed on malignant myeloid blasts and marrow stromal compartments in patients with MDS, CMML, and AML treated with HMA (36). In preclinical models, decitabine treatment has been seen to promote expansion of progenitor exhausted CD8+ T cell states characterized by effector activation (89). These findings have provided the rationale for multiple investigators to combine HMAs with immune checkpoint inhibitors with heterogenous results (Section 7.1).

### 5.3 Effects of HMAs on TCR repertoire and clonality

TCR repertoires in myeloid malignancies are frequently contracted at baseline, with multiple studies demonstrating hyperexpanded clonotypes with reduced diversity in MDS BMs (90, 91). The degree of TCR repertoire abnormality also appears to correlate with disease severity, with profoundly abnormal TCR repertoires characterized by advanced IPSS stage, increased WT-1 expression, and higher blast counts (50).

HMAs have been trialed alone or in combinatorial approaches to reshape TCR diversity. In a Cancer Genome Atlas methylation analysis, T cells from MDS/AML patients demonstrated hypermethylation patterns at multiple TCR-associated loci compared to healthy donors, with decitabine treatment subsequently demethylating these loci (92). Deep CDR3B sequencing suggests that HMA-associated remodeling is primarily defined by the emergence of new clonotypes during treatment. In deep sequencing of 11 MDS patients treated with HMA, overall clonality richness and diversity did not differ significantly between responders and nonresponders. However, responders developed new CDR3 sequences and had emergence of new T cell populations.

Clinically, a broader baseline TCR diversity also appeared to affect later immune remodeling and responses to HMA. In the RAS-AZIC trial of 51 elderly AML patients, elevated pretreatment BM T cell diversity and increased diversity after a single treatment cycle of azacitidine were both associated with significantly improved overall survival rates at 24 months (83.3 vs 60.6%) (93). Similarly, in a study of 55 patients with MDS, responders to treatment with immunotherapy and HMAs had a higher baseline TCRB diversity compared to non-responders and additionally had a more significant increase in TCRB clonality and number of novel T cell clones (94). These findings suggest that baseline oligoclonal TCR contraction may leave fewer naïve T cells available for HMA-induced antigen responses following treatment. Supporting this concept, a phase I trial combining decitabine with immunotherapy and NY-ESO-1 antigen vaccination in 5 patients with MDS generated NY-ESO-1 specific CD4+ T cell responses in 4 of 5 treated patients (95), but lacked a concomitant CD8+ T cell response. These patients were then seen to have significantly reduced dendritic-cell populations and lower CXCL9/CD80 expression at baseline compared to healthy donors. These observations are consistent with a tumor microenvironment characterized by APC dysfunction/impaired T-cell recruitment and provide a rationale for HMA-based combinatorial approaches that bypass or stimulate APC-dependent pathways.

### 5.4 Effects of HMA on NK cell function

Hypomethylating agents have been observed to upregulate cell-surface NKG2D ligands on myeloid blasts, restoring susceptibility to NK-mediated cytolysis (96). Low-dose azacitidine also demethylates the killer immunoglobulin-like receptor (KIR) loci which are typically epigenetically silenced in dysfunctional MDS NK cells, thereby restoring KIR diversity, IFN-γ production, and degranulation of NK cells in MDS (97).

## 6 Therapeutic T cell suppression in MDS

Hypoplastic MDS provides additional evidence for pathogenic T-cell activity. Several studies have shown that cytotoxic T-cell populations can inhibit hematopoietic progenitor growth. Oligoclonal CD8-positive T-cell expansions in MDS with erythroid hypoplasia suggests that T cells may be responsible for suppression of erythropoiesis and capable of inhibiting marrow hematopoeisis (98, 99). This concept is further reinforced by the responsiveness of hypoplastic MDS patients to immunosuppressive therapy. Antithymocyte globulin and cyclosporine have produced hematologic responses in subsets of MDS patients with features of immune-mediated marrow failure (17, 100, 101). Small clinical trials suggest younger age, hypocellular MDS, HLA-DR15 positivity and the presence of trisomy 8 correlate with better outcomes. In a large, international, retrospective cohort of 207 patients treated with IST the overall response rate was 48.8% and red blood cell transfusion-independence was achieved in 30% (102). Multivariate analysis revealed no predictors of response, and only hypocellular marrow (<20%) was associated with achievement of transfusion independence. Overall survival was better for patients who achieved a response and the only variable significantly associated with improved survival in multivariate analysis was low bone marrow blast count (<5% vs >5%). Despite these relatively positive results, IST remains a little-used strategy for patients with MDS, presumably because ATG requires inpatient administration and monitoring for toxicities whereas newer agents for low risk MDS are administered outpatient with relatively few toxicities.

In another study, immunosuppressive therapy responsive patients with MDS showed T-cell responses directed against WT1 (103). Chamuleau et al. demonstrated that activated lymphocytes, increased effector T-cell populations with cytotoxic profiles, TCR Vβ skewing, and autologous cytotoxicity directed against aberrant hematopoietic precursor cells in lower-risk MDS (104), suggesting that leukemia-associated antigens may participate in the immune-mediated marrow failure phenotype.

## 7 Targeting immune checkpoint pathways in MDS

Increased expression of immune escape surface antigens on leukemic cells have been well described in MDS. As such, mechanisms to promote T-cell mediated immune control through checkpoint inhibition has attracted significant attention as a therapeutic strategy in MDS, though clinical responses have remained variable.

### 7.1 PD-1/PD-L1

In MDS, expression of PD-L1 and PD-L2 is increased on malignant HSPCs compared to normal hematopoietic progenitors, with expression levels increasing following HMA treatment and with higher risk disease (36, 105). Several early phase trails have demonstrated modest response rates, including a phase II trial of azacitidine with pembrolizumab in patients with intermediate- higher-risk MDS; ORR reached 76% with an 18% CR rate in 17 previously untreated patients, whereas the HMA-failure cohort demonstrated an ORR of 25%, with a 5% CR rate and mOS of 5.8 months (36). In a recent phase II study of azacitidine combined with nivolumab, ipilimumab, or both ICIs in treatment naïve higher risk MDS, triplet therapy showed no improvement in OS or EFS despite increased hematologic and immune-related toxicity, with significantly higher rates of hospitalization and post-transplant mortality (106). Together, these findings suggest that unselected HMA and ICI combinations are unlikely to achieve broad benefit in MDS, but that biomarker-stratified enrollment may define future trials.

In a phase I/II trial of guadecitabine combined with atezolizumab, 33 patients with HMA-relapsed or HMA-refractory MDS/CMML achieved an overall response rate of 33%, median overall survival of 15.1 months, with grade 3 or lower immune-related adverse events in 36%. Importantly, patients with *ASXL1* co-mutated T lymphocytes and myeloid cells demonstrated disproportionately favorable (2 with CR) and durable response rates compared to WT (mOS 48.9 vs 16.4 months, unpublished long-term follow-up data). This association remained significant after adjustment for age, sex, diagnosis, and number of prior regimens (65), suggesting that intrinsic epigenetic T-cell priming may be a prerequisite to checkpoint inhibition response in MDS.

Mechanistic support for this potential biomarker of ICI response is provided in the 2024 Science study by Kang and colleagues (107). Using murine models of chronic antigen exposure and tumor immunotherapy, they showed that disruption of *DNMT3A*, *TET2*, or *ASXL1* in CD8-positive T cells preserved a progenitor-exhausted, stem-like T-cell population for more than one year without malignant transformation. Specific investigation of *ASXL1* demonstrated that *ASXL1* disruption preserved TCF1-positive stem-like CD8 T cells, maintained self-renewal capacity, allowed differentiation into functional effectors, and synergized with anti–PD-L1 therapy in tumor models. This provides a mechanistic explanation for why *ASXL1* mutated T cells might be favorable in the setting of checkpoint blockade. *ASXL1* disruption altered the polycomb repressive deubiquitinase pathway and histone H2A lysine 119 ubiquitination, preserving chromatin accessibility at stemness associated loci such as TCF7 and LEF1. Functionally, *ASXL1*-disrupted CD8 T cells retained both stem-like properties and effector potential, a combination that is particularly relevant to PD-1/PD-L1 blockade, where durable benefit depends on a pool of checkpoint-responsive, self-renewing T cells rather than terminally exhausted effectors alone. Analyzed T cells from long-term surviving MDS patients with known ASXL1 disruption after anti–PD-L1 therapy showed that *ASXL1* VAF increased after treatment in PD-1-low CD4-positive T cells and in CD8-positive T-cell subsets. This supports the model in which *ASXL1*-disrupted T cells may have a selective survival or expansion advantage during PD-L1 blockade. These data suggest that *ASXL1* mutation in T cells may identify a subset of patients whose T cells are unusually capable of persisting, expanding, and mediating benefit during checkpoint-based therapy.

### 7.2 TIM-3

TIM-3 ligands have been expressed on multiple cell types in the innate and adaptive immune compartment, including in exhausted CD8+ T cells, NK cells, MDSCs, and leukemic stem cells. Expression is enriched on leukemic stem cells compared to normal HSPCs, and higher expression levels have been correlated to higher risk disease in MDS and increased risk of progression to AML (108).

In MDS, TIM-3 signaling is mediated primarily through its principal ligands galectin-9 and CEACAM1. TIM-3/Gal-9 engagement suppressing IFN-γ production, promotes apoptosis of activated CD8+ T cells, and contributes broadly to T-cell dysfunction in the marrow (109). Gal-9 secretion by MDSCs and leukemic cells have additionally been implicated in the depletion of cytotoxic T cell populations. TIM-3/CEACAM1 interactions appear to be more closely linked to maintenance of dysfunctional exhausted T cell states, with co-expression defining the most severely impaired effector subsets and most resistant to single checkpoint reinvigoration (110).

Targeting this pathway has proven difficult in clinical trials despite early promise. Sabatolimab, an anti-TIM-3 IgG4 monoclonal antibody, initially demonstrated encouraging results in a phase Ib trial of 51 patients with high-/very high-risk MDS, with a median duration of response of 16 months and an ORR of 57% (111). However, the subsequent randomized phase II STIMULUS-MDS1 (NCT03946670) and phase III STIMULUS-MDS2 (NCT04266301) failed to demonstrate a survival benefit over azacitidine. Novartis has since discontinued development of sabatolimab in MDS, but it remains unclear whether the benefit observed in the phase Ib data reflects a responsive subset as neither trial evaluated TIM-3 expression density, marrow immune architecture, or evaluation of T cell genotypes or phenotypes.

## 8 Personalized therapy based on T lymphocyte mutations

Personalized therapy directed at T-lymphocytes is a provocative concept in MDS. A review of clinical trials targeting T cells is provided in **Table 2**. At present, no approved MDS therapy is selected based on T cell behavior or involvement by the clone. Standard treatment selection remains driven by disease risk, cytogenetics, and clinical data. However, with the growing recognition that T cells in MDS may be clonally involved, mutations detected in T lymphocytes may eventually guide therapeutic selection, particularly when integrated with conventional MDS risk stratification. CD3-positive T cells from patients with MDS can be derived from the malignant clone. However, mutations such as *TET2*, *DNMT3A*, and *ASXL1* may also be T-cell intrinsic alterations (112).

**Table 2:** Clinical trials of T-lymphocyte and NK-cell directed therapies in MDS.

Agent or regimen	Trial and phase	Immune target and target cell	Setting	Key efficacy and survival outcomes	Ref
Immunosuppressive therapy					
ATG and cyclosporine	International retrospective cohort, 207 patients	Pathogenic cytotoxic T cells	Lower-risk and hypocellular MDS	ORR 48.8% and RBC transfusion independence 30%. Hypocellular marrow under 20% predicted transfusion independence. Response and blast count under 5% associated with better overall survival.	(100)
Differentiation and MDSC-directed therapy					
Decitabine plus ATRA versus decitabine	Randomized trial, 277 MDS-EB patients	Myeloid differentiation and MDSC maturation	Higher-risk MDS with excess blasts	ORR 78% versus 51% for decitabine alone.	(46)
Tamibarotene	SELECT-MDS-1, phase III	RARα agonist, myeloid differentiation	Higher-risk MDS	Negative trial with no benefit over control.	(48)
AMV564	Early phase	CD33 and CD3 bispecific that depletes MDSCs and activates cytotoxic T cells	MDS	Improved hematopoiesis after MDSC depletion. Development discontinued after company closure.	(49)
Checkpoint monotherapy					
PD-1 or PD-L1 blockade as monotherapy	Early phase	PD-1 or PD-L1 on T cells	MDS, including after HMA failure	Uniformly modest responses with minimal single-agent activity.	(35)
Hypomethylating agent plus immune checkpoint inhibitor					
Azacitidine plus pembrolizumab	Phase II	PD-1 on CD8 T cells	Treatment-naive and HMA-failure MDS	Treatment-naive ORR 76% and CR 18% in 17 patients. HMA-failure ORR 25%, CR 5% and median OS 5.8 months.	(35)
Azacitidine plus nivolumab, ipilimumab, or both	Phase II	PD-1 and CTLA-4 on T cells	Treatment-naive higher-risk MDS	Triplet therapy gave no improvement in OS or EFS, with increased immune-related toxicity, hospitalization and post-transplant mortality.	(106)
Guadecitabine plus atezolizumab	Phase I/II	PD-L1, with lineage-resolved T-cell sequencing	HMA-relapsed or refractory MDS and CMML	ORR 33%, median OS 15.1 months and immune-related adverse events in 36%. ASXL1 co-mutation in the T-cell compartment associated with prolonged survival.	(63)
Anti-TIM-3					
Sabatolimab	Phase Ib with 51 patients, then STIMULUS-MDS1 phase II and STIMULUS-MDS2 phase III	TIM-3 on exhausted T cells, NK cells and leukemic stem cells	High and very high-risk MDS	Phase Ib ORR 57% and median duration of response 16 months. Randomized STIMULUS-MDS1 and STIMULUS-MDS2 showed no survival benefit over azacitidine. Development discontinued.	(110)

ATG, antithymocyte globulin; ATRA, all-trans retinoic acid; CMML, chronic myelomonocytic leukemia; CR, complete remission; EFS, event-free survival; HMA, hypomethylating agent; MDS-EB, MDS with excess blasts; MDSC, myeloid-derived suppressor cell; ORR, overall response rate; OS, overall survival; RARα, retinoic acid receptor alpha; RBC, red blood cell.

Time to next treatment was not consistently reported across these trials and is therefore not tabulated. Where reported, survival and duration-of-response outcomes are shown.

Agents and regimens discussed in the review, grouped by immune strategy, with target cell population, disease setting, and key efficacy and survival outcomes. Reference numbers correspond to the manuscript reference list.

The most common example of T cell directed therapy in MDS is when large granular lymphocytic leukemia (LGL) like T-cell expansions co-exist. T-LGL is a chronic clonal cytotoxic T-cell disorder associated with neutropenia, anemia, pure red cell aplasia, autoimmune disease, and bone marrow failure. Coincidence of T-LGL proliferations and MDS has been recognized for decades, and multiple series have shown that T-LGL expansions can coexist with myeloid neoplasia (113–116). In fact, T-LGL proliferations in MDS are associated with marrow hypocellularity and lineage hypoplasia, particularly erythroid hypoplasia. The discovery of recurrent *STAT3* and *STAT5B* mutations in T-LGL leukemia created a molecular basis for identifying pathogenic cytotoxic T-cell clones. STAT3 mutations were reported in a substantial fraction of patients with T-LGL leukemia that are associated with cytopenias (117). In an MDS patient, detection of a *STAT3* or *STAT5B* mutated cytotoxic T-cell clone, the T-cell clone may be the driver of cytopenias rather than the myeloid clone. In such patient’s, treatment with immunosuppression or LGL-directed therapy may be more appropriate to treat the underlying pathology (118).

*TET2* requires explicit discussion because it is one of the most frequent mutations in MDS and clonal hematopoiesis but can also have lymphoid or NK-cell relevance. In MDS, a *TET2* mutation detected in bulk marrow sequencing usually supports a myeloid clonal process. Prior studies have associated higher-abundance *TET2* mutations with increased response rates to hypomethylating agents, particularly when *ASXL1* is not co-mutated, although subsequent datasets have been less uniform (119, 120). However, *TET2* cannot be regarded as selectively myeloid. *TET2* mutations are common in chronic NK-cell large granular lymphocytic leukemia and chronic lymphoproliferative disorders of NK cells (121, 122). Disruption of *TET2* in T cells has been associated with enhanced persistence and therapeutic efficacy of CD19-directed CAR T cells, while *DNMT3A* deletion has been shown to prevent CAR T-cell exhaustion and enhance antitumor activity. These observations do not establish *TET2* or *DNMT3A* mutation as clinically actionable biomarkers in MDS yet, but they highlight epigenetic regulator mutations as critical in T-cell durability, memory formation, exhaustion resistance, and response to immunotherapy (50, 123).

The most recent clinical example in MDS is the discovery of *ASXL1* mutations involving the T-cell compartment in responders to immune checkpoint therapy. This creates a clinically important paradox. Myeloid *ASXL1* mutation is generally considered adverse in MDS/CMML, whereas T-cell-involved *ASXL1* mutation may be favorable in the specific context of PD-1/PD-L1-directed therapy. The same gene may therefore carry opposite implications depending on cellular lineage. In such situations, bulk marrow sequencing is insufficient for immune-personalized therapy. A bulk *ASXL1* mutation result cannot distinguish myeloid-only disease biology from shared myeloid/T-cell involvement. In future trials, *ASXL1* should be reported not only as mutated or wild type, but by lineage; ideally PD-1-high versus PD-1-low T-cell fractions would also be examined when checkpoint therapy is being studied.

As more T cell lineage mutations and roles in MDS are elucidated, algorithms in T-lymphocyte mutations can be developed. *STAT3* or *STAT5B* mutation in an expanded cytotoxic T-cell/LGL population should raise concern for an immune clone contributing to cytopenias and may support LGL-directed or other immunosuppressive therapy. *TET2* mutation should trigger lineage assignment, as it may represent myeloid MDS/CHIP, NK-LGL/CLPD-NK biology, or an ancestral multilineage lesion. *DNMT3A* and *TET2* alterations in T cells are not yet validated clinical biomarkers in MDS, but they remain relevant due to their contributions in T-cell exhaustion, persistence, and engineered T-cell function. Presence of *ASXL1* mutations in T cells is currently the most compelling candidate biomarker for checkpoint-based therapy in MDS/CMML, particularly when present at higher T-cell VAF.

Prospective validation will be essential. Future MDS immunotherapy trials should incorporate sorted-cell sequencing at baseline and on treatment. They should also include TCR sequencing, flow cytometric assessment of exhaustion and stemness markers, checkpoint ligand analysis, and tracking of T-cell VAF. For *ASXL1* specifically, trials should define whether there is a clinically meaningful T-cell VAF threshold, whether CD8-positive involvement is more predictive than bulk CD3-positive involvement, whether *ASXL1*-mutated T cells expand after PD-1/PD-L1 blockade, and whether expansion correlates with survival, response, or immune-related toxicity.
